# Beyond Trophic Factors: Exploiting the Intrinsic Regenerative Properties of Adult Neurons

**DOI:** 10.3389/fncel.2019.00128

**Published:** 2019-04-05

**Authors:** Arul Duraikannu, Anand Krishnan, Ambika Chandrasekhar, Douglas W. Zochodne

**Affiliations:** Division of Neurology, Department of Medicine, and Neuroscience and Mental Health Institute, University of Alberta, Edmonton, AB, Canada

**Keywords:** peripheral nerve, RhoA, Netrin/Unc5H, PTEN, RB1, BRCA1, APC/β-catenin

## Abstract

Injuries and diseases of the peripheral nervous system (PNS) are common but frequently irreversible. It is often but mistakenly assumed that peripheral neuron regeneration is robust without a need to be improved or supported. However, axonal lesions, especially those involving proximal nerves rarely recover fully and injuries generally are complicated by slow and incomplete regeneration. Strategies to enhance the intrinsic growth properties of reluctant adult neurons offer an alternative approach to consider during regeneration. Since axons rarely regrow without an intimately partnered Schwann cell (SC), approaches to enhance SC plasticity carry along benefits to their axon partners. Direct targeting of molecules that inhibit growth cone plasticity can inform important regenerative strategies. A newer approach, a focus of our laboratory, exploits tumor suppressor molecules that normally dampen unconstrained growth. However several are also prominently expressed in stable adult neurons. During regeneration their ongoing expression “brakes” growth, whereas their inhibition and knockdown may enhance regrowth. Examples have included phosphatase and tensin homolog deleted on chromosome ten (PTEN), a tumor suppressor that inhibits PI3K/pAkt signaling, Rb1, the protein involved in retinoblastoma development, and adenomatous polyposis coli (APC), a tumor suppressor that inhibits β-Catenin transcriptional signaling and its translocation to the nucleus. The identification of several new targets to manipulate the plasticity of regenerating adult peripheral neurons is exciting. How they fit with canonical regeneration strategies and their feasibility require additional work. Newer forms of nonviral siRNA delivery may be approaches for molecular manipulation to improve regeneration.

## Introduction

Favorable outgrowth of peripheral nervous system (PNS) axons after injury is often considered in comparison with that of the central nervous system (CNS) where severe barriers, even for limited outgrowth occur. In the clinical context, however, there is striking evidence for its inadequacy. Functional recovery from trauma or disease to the PNS is slow, incomplete and complicated by neuropathic pain. Long target distances, gaps from nerve transection and delayed regrowth into distal nerve territories add substantial and additional barriers to regrowth (Zochodne, 2012). Patients with severe, nominally reversible peripheral nerve disorders such as Guillain-Barre syndrome or vasculitis have prolonged, if not permanent deficits. Similarly, large proximal nerve lesions such as those at the brachial plexus or high sciatic nerve rarely recover completely. There are both intrinsic and extrinsic factors that act as barriers to the successful regrowth of neurons.

**Figure 1:**
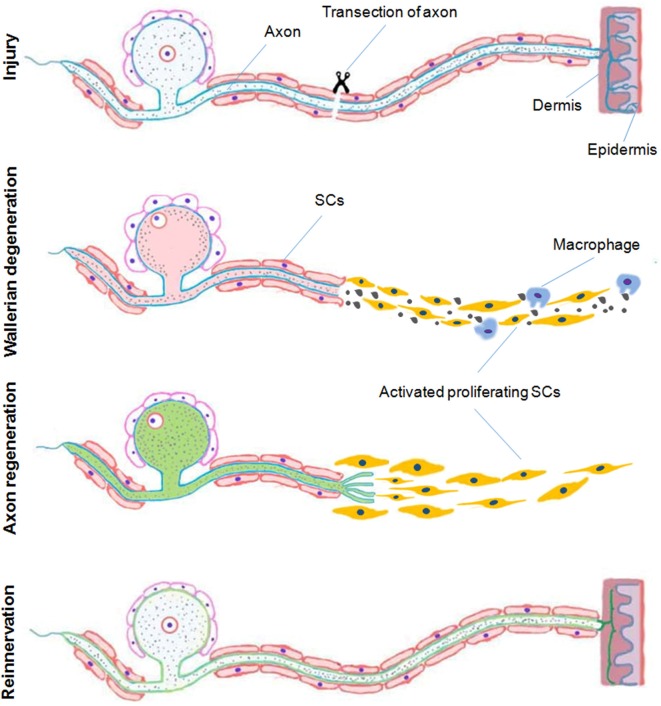
A schematic representation of Wallerian degeneration and regeneration after peripheral nervous system (PNS) injury. In the peripheral nerve following and acute transection axonal injury, Wallerian degeneration, which takes place during few days after injury, is characterized by proliferation and activation of Schwann cells (SCs), recruitment of inflammatory cells including macrophages and dissolution and clearance of the myelin sheath and debris. Proliferating SCs respond by organizing themselves providing a pathway for newly sprouting axons during regeneration and later remyelination associated with reinnervation of the distal target tissue (Duraikannu, original illustration).

Following acute axonal injury to peripheral nerves, a series of active molecular events that degrade the distal axon develop. “Wallerian” degeneration refers to these degenerative events in distal nerve stumps strictly after transection whereas “axonal degeneration (AxD)” (or “Wallerian-like degeneration”), is a broader term that encompasses all forms of irreversible axon injury. While AxD is initiated immediately after axon injury, a series of morphological changes soon follow. There is fragmentation of axons, dissolution of their neurofilament scaffolds, proliferation and activation of Schwann cells (SCs), recruitment of inflammatory cells including macrophages, and dissolution then clearance of the myelin sheath and axon debris (Waller, 1850; Ide, 1996; Burnett and Zager, 2004; Chen et al., 2007; Zochodne, 2008; Figure 1). Following axonal degeneration, proliferating SCs organize themselves into bands of Bungner. These are tubular collections of both SC and basement membrane that serve as both guideposts and channels for newly sprouting axons. These peripheral events accompany changes in neuron perikarya and associated satellite glial cells. For example perineuronal satellite cells which surround nerve cell bodies in sensory and autonomic ganglia, begin to proliferate within a 2–3 days after peripheral axon injury accompanied by local proliferation of resident macrophages, both constituting a population of dorsal root ganglia (DRG) recycling cells (DRCCs) (Wong and Mattox, 1991; Sulaiman and Gordon, 2000; McKay Hart et al., 2002; Zochodne, 2008; Krishnan et al., 2018a).

At most steps during regeneration, there is unexpected axon hesitation. For example, axon regrowth from proximal nerve stumps after transection is delayed, slow and staggered. “Pioneer” axons, which resemble those of development are the first to emerge, but almost always follow leading SCs and their processes (Chen et al., 2005; McDonald et al., 2006). Navigation of axons across gaps that form between the proximal and distal stump is challenging and it is estimated that only one in 10 axons traverse it (Witzel et al., 2005). Distal nerve stumps over time also become inhospitable to hosting regrowing axons. This is contributed to by loss or atrophy of SCs, declines in their elaboration of SC-derived growth factors and declines in the local vascular supply (Sulaiman and Gordon, 2000; Hoke et al., 2001, 2002).

Here, we discuss why peripheral neurons do not grow as robustly as assumed. We begin with a discussion of growth factors that support the PNS, but shift our emphasis to intrinsic controls of growth within neurons and the potential to manipulate novel molecular pathways to enhance growth after injury (Table 1).

**Table 1:** A selected listing of approaches discussed in this review that promote axonal regeneration.

**Neurotrophic factors**
Nerve growth factor (NGF)
Brain derived neurotrophic factor (BDNF)
Neurotrophin-3 (NT-3) and neurotrophin-4/5 (NT-4/5)
Ciliary neurotrophic factor (CNTF)
Leukemia inhibitory factor (LIF)
Oncostatin M (OSM)
Glial cell line-derived neurotrophic factor (GDNF)
Hepatocyte growth factor (HGF)
Cardiotrophin-1 (CT-1)
Neurturin (NRTN)
Artemin (ARTN)
Persephin (PSP)
Bone morphogenetic proteins (BMPs)
Epidermal growth factor (EGF)
Fibroblast growth factor (FGF)
Osteopontin (OPN)
Insulin
**Electrical stimulation**
**Developmental molecules in regeneration**
Unc5H inhibition
**Growth cone manipulation**
Inhibition of RHOA-ROK
**Influence of tumor suppressor pathways during regeneration**
Phosphatase and tensin homolog (PTEN)
Retinoblastoma 1 (Rb1)
APC-β-catentin pathway

## Extrinsic Support of Regeneration Through Growth Factors

The discovery over the past 50 years that extrinsic molecules can influence the behavior of neurons, either during development or during adulthood was a remarkable advance. The prototypic growth factor was nerve growth factor (NGF) discovered in mouse submaxillary glands and snake venom that supported robust outgrowth of branches from sympathetic neurons. For its discovery, the Nobel prize in Medicine and Physiology was awarded to Drs Levi-Montalcini and Cohen in 1986. NGF was the first of a family of extrinsic growth factors termed neurotrophins. Several of these are discussed below (Table 1). Beyond this family, an expanded repertoire of growth factors is now available for improving the outcome of peripheral nerve trunk injury but their practical application remains problematic. Each axon subtype has a limited receptor deployment available to a given growth factor, requiring cocktails to support all classes of fibers in a nerve trunk. There also remain issues with access, timing of such delivery, where to administer them and their overall stability (Kemp et al., 2007).

### Neurotrophin Family Members

#### Nerve Growth Factor

NGF supports the survival and subsequent differentiation of sensory and sympathetic neurons of the PNS (Levi-Montalcini, 1950, 1987). In the CNS, NGF plays a role in the neurodevelopment and ongoing maintenance of basal forebrain cholinergic neurons (Dreyfus, 1989), septo-hippocampal pathways, hippocampal neurons and cortical neurons (Zhang et al., 1993; Culmsee et al., 1999a,b; Kume et al., 2000). NGF mediates its trophic effect by binding to specific receptors, tropomyosin-receptor kinase A (TrkA) and p75 neurotrophin receptor (p75^NTR^) (Hempstead et al., 1991; Chao, 2003; Huang and Reichardt, 2003). When p75^NTR^ is activated in the absence of Trk ligation, however, it promotes apoptosis of neurons. In the presence of Trks, p75^NTR^ enhances neurotrophin responses (Kaplan and Miller, 1997). After sciatic nerve injury, NGF receptor expression increased in motor neurons at spinal levels L4-L6 where it reached a maximum level between 1 and 7 days, and normalized to baseline by day 30 (Rende et al., 1992a; DiStefano and Curtis, 1994). NGF is also increased in injured distal nerve stumps (Richardson and Ebendal, 1982; Heumann et al., 1987) and it probably protects adult rat DRG neurons from retrograde cell atrophy and death following axotomy injury (Rich et al., 1987). NGF treatment to pre-injured sensory neurons *in vitro*, derived from young (3 months) and old (26 months) mice supports neurite outgrowth (Horie et al., 1991). Similarly, delayed administration of NGF to the sciatic nerve 3 weeks after transection restores high-affinity NGF receptor density and partially restores neuronal volume (Verge et al., 1989). Topical application of NGF (1 μg) into a nerve crush site increases motor nerve conduction velocity (MNCV), and numbers of regenerating myelinated fibers (RMFs) in rat sciatic nerves (Chen and Wang, 1995). Similar benefits accrue when NGF is given by silicone chamber to injured nerves (Derby et al., 1993; Santos et al., 2017) although gradients are difficult to establish (Kemp et al., 2007).

#### Brain Derived Neurotrophic Factor

Perhaps the best characterized and most promising neurotrophin family member is brain derived neurotrophic factor (BDNF). BDNF has essential roles in neuronal survival, growth and differentiation during development, and synaptic plasticity in adult peripheral neurons (Thoenen, 1995; Huang and Reichardt, 2001). As such, BDNF has been a potential candidate to promote nerve regeneration. After injury, the BDNF mRNA expression is increased in DRG, SCs and muscle fibers (Zhang et al., 2000; Fukuoka et al., 2001; Kobayashi et al., 2008). Enhanced BDNF protein expression has also been identified in DRG neurons after spinal dorsal horn injury (Fukuoka et al., 2001; Miletic and Miletic, 2002; Geng et al., 2010). More importantly, BDNF and its receptor TrkB (affinity tyrosine kinase receptor B) are robustly expressed in both DRG sensory, and spinal motor neurons (Foster et al., 1994; Hammarberg et al., 2000) after sciatic nerve injury. Specifically, BDNF was expressed prominently in both small and medium size DRG neurons following nerve injury (Cho et al., 1998; Kashiba and Senba, 1999). Sensory-neuron derived BDNF is also transported in the anterograde direction, appearing at the injured nerve site. The levels of BDNF decline over weeks following the injury (Zhou and Rush, 1996; Tonra et al., 1998). Furthermore, BDNF and TrkB receptors are expressed in different muscles to coordinate muscle innervation and the functional differentiation of neuromuscular junctions (Chevrel et al., 2006). In contrast, following nerve injury deprivation of endogenous BDNF showed attenuated axon outgrowth, and reduced myelinated axon repopulation and regeneration (Song et al., 2008). Treatment with exogenous BDNF to peripheral nerves is transganglionically transported within the spinal cord (Curtis et al., 1998) and as expected, increased recovery after spinal cord injury (Song et al., 2008). In addition, single intrathecal injections of BDNF effectively produce long-lasting thermal hyperalgesia and tactile allodynia in normal mice and may play an important role in chronic pain syndromes (Yajima et al., 2002; Nijs et al., 2015; Sikandar et al., 2018). Delivery of BDNF to the hindpaw or sciatic nerve improves locomotion recovery after contusion injury (Song et al., 2008). Therefore, given these data, there is a reason to believe that BDNF may offer therapeutic benefits for peripheral nerve regeneration.

#### Neurotrophins-3 and 4/5

Neurotrophin-3 (NT-3) and neurotrophin-4/5 (NT-4/5), additional members of neurotrophin family, support nervous system development, survival, differentiation, and repair (Yamamoto et al., 1996). In the CNS, NT-3 prevents degeneration of noradrenergic (Arenas and Persson, 1994) and dopaminergic neurons (Hyman et al., 1994). NT-3 with or without BDNF improves axonal regeneration after spinal cord injury (Schnell et al., 1994; Xu et al., 1995; Ramer et al., 2001; Liu et al., 2016; Keefe et al., 2017). NT-4/5 stimulates axonal branching from regenerating retinal ganglion cells (RGCs; Sawai et al., 1996). In the PNS, NT-3 promotes neurite outgrowth and survival in peripheral sensory, motor and sympathetic neurons (Rosenthal et al., 1990; Henderson et al., 1993). NT-3 binds to the TrkC receptor (Katoh-Semba et al., 1996) mainly in large sensory neurons (Zhou and Rush, 1993). NT-4 at the site of sciatic nerve injury increases axon numbers, axonal diameter, myelin thickness and sciatic function index (Yin et al., 2001). NT-4/5 prevents the cell death of embryonic rat spinal motor neurons *in vitro* (Henderson et al., 1993). NT-3 and NT-5 stimulate functional reinnervation of skeletal muscle (Braun et al., 1996).

### Other Extrinsic Growth Factors

#### Ciliary Neurotrophic Factor

Ciliary neurotrophic factor (CNTF) is derived from parasympathetic cholinergic neurons and is highly expressed in embryonic chick eye (Helfand et al., 1976; Adler et al., 1979; Barbin et al., 1984), adult rat peripheral nerve axons, SCs and spinal nerve roots (Williams et al., 1984; Manthorpe et al., 1986; Millaruelo et al., 1986; Rende et al., 1992b). In the CNS, CNTF is expressed in the optic nerve, mainly in astrocytes (Stöckli et al., 1991). Intravitreal injection of recombinant CNTF increased RGC survival and axon regeneration (Müller et al., 2009). In nerve, CNTF expression appears to be downregulated in the distal stump after injury site but recovered within SCs during regeneration (Williams et al., 1984; Sendtner et al., 1992). CNTF was detected in both small and large subpopulation neurons and regenerating neurites *in vitro* (Sango et al., 2007). Exogenous CNTF prevents degeneration of motor neurons after facial nerve lesions in neonatal rats (Sendtner et al., 1990). In addition, injured nerve sites exposed to recombinant human CNTF had greater numbers of regrowing axons in the distal stumps after injury (Sahenk et al., 1994). Topical application of CNTF to injured sciatic nerves resulted in higher MNCV indicating larger, more mature axons and higher compound muscle action potential amplitudes of the anterior tibial muscle (Zhang et al., 2004b). Likewise, CNTF null (−/−) mutant mice have reductions in axon diameter, had myelin sheath disruption, and demonstrated loss of axon-SC cell architecture at nodes of Ranvier (Gatzinsky et al., 2003). Exposure to CNTF enhanced neurite outgrowth of dissociated adult sensory neurons *in vitro* (Saleh et al., 2013). Finally, CNTF treatment improved sensory nerve regeneration after crush injury in diabetic rats (Mizisin et al., 2004).

#### Leukemia Inhibitory Factor

Leukemia inhibitory factor (LIF) supports the neurodevelopment of sensory neurons from the neural crest (Murphy et al., 1991). In the mouse DRG (embryos), LIF supports the survival of NGF non-responsive neurons and regulates sensory development *in vivo* (Murphy et al., 1993). LIF supports the survival of sensory neurons after sciatic nerve injury in rat pup. Interestingly, sciatic nerve injury induced LIF expression in the distal and proximal stumps and in denervated muscle fibers (Curtis et al., 1994; Kurek et al., 1996). Injured sciatic nerves treated with a silicone cuff containing LIF increased the recovery of muscle contraction, conduction velocity, myelinated fiber number and diameter (Tham et al., 1997). LIF deleted nerve segments were less supportive of axonal outgrowth (Ekström et al., 2000) and LIF knockdown mouse had impaired muscle regeneration (Kurek et al., 1997). In the CNS, deletion of LIF expression was associated with impaired axon sprouting of cultured RGC neurons *in vitro* and delayed recovery after optic nerve injury *in vivo* (Ogai et al., 2014).

#### Oncostatin M

Oncostatin M (OSM), a neuroprotective cytokine of the interleukin-6 family (Taga and Kishimoto, 1997; Heinrich et al., 1998; Senaldi et al., 1999) attenuates neuronal neuron death (Weiss et al., 2006). OSM induces signals through glycoprotein 130 (gp130) and the OSM-specific β subunit receptor complex (Mosley et al., 1996). In the PNS, OSMR beta was expressed in small caliber non-peptidergic neurons of the dorsal root and trigeminal ganglia (Tamura et al., 2003; Morikawa et al., 2004; Morikawa, 2005). OSM mRNA expression in the nerve increased rapidly up to 14 days following injury (Ito et al., 2000). Interestingly, subcutaneous injection of OSM into the hind paw of *C57BL6J* wild type mice was associated with a reduction of paw withdrawal latencies to heat stimulation (Langeslag et al., 2011), indicating support for axon repair (Ito et al., 2000).

#### Glial Cell Line-Derived Neurotrophic Factor

Glial cell line-derived neurotrophic factor (GDNF), belongs to the transforming growth factor-β (TGF-β) superfamily (Tomac et al., 1995), and enhances the survival and morphological differentiation of midbrain dopaminergic neurons (Lin et al., 1993). Recombinant GDNF promotes the survival of motor neurons (Chen et al., 2000), sympathetic neurons and enhanced neurite outgrowth in embryonic chick sympathetic neurons (Trupp et al., 1995). Local application of GDNF to the injured neonatal facial nerve prevented retrograde motor neuron loss and atrophy (Yan et al., 1995). After injury, GDNF and its receptor, GFRα-1, mRNA levels increased in sciatic nerve (Trupp et al., 1995) distal stumps. Interestingly, GFRα-1 was also increased in the DRG ipsilateral to the nerve injury (Hoke et al., 2000). GDNF mRNA and protein expression was upregulated in SCs 48 h after injury and declined to basal levels by 6 months of denervation. GFRα-1 and GFRα-2 mRNAs were increased only after GDNF upregulation and remained elevated as late as 6 months (Hoke et al., 2002). Pre-conditioning injury of cultured DRG neurons *in vitro* treated with exogenous GDNF increased neurite outgrowth (Mills et al., 2007). In addition, GDNF induced neurite outgrowth and upregulation of galectin-1 (GAL-1) through the RET/PI3K pathway in DRG sensory neurons *in vitro* (Takaku et al., 2013). GDNF also induced directional turning of adult neuron growth cones but only did so in the company of hepatocyte growth factor (HGF) or a phosphatase and tensin homolog (PTEN) inhibitor (Guo et al., 2014). GDNF also influences SC function (Zhang et al., 2009). In contrast, lentiviral vector-mediated GDNF overexpression for 16 weeks increased GDNF expression at regenerating sites but impaired long-distance nerve regeneration (Eggers et al., 2008; Tannemaat et al., 2008; Ortmann and Hellenbrand, 2018). Finally, GDNF may offer analgesia in animal models of neuropathic pain (Boucher et al., 2000).

#### Hepatocyte Growth Factor

HGF, was initially identified as a mitogen for hepatocytes (Nakamura et al., 1984, 1989). HGF interacts with c-Met receptor tyrosine kinase (Bottaro et al., 1991). In normal DRG, c-Met receptor was expressed in small and medium-size neurons and to a lesser extent in large-size neurons. However, following sciatic nerve ligation (SNL) c-Met expression increased after injury (Zheng et al., 2010). Mutations in the HGF receptor (Met tyrosine kinase), show abnormal limb innervation correlated with reductions of muscle fibers of mouse embryos (Maina et al., 1997). In normal DRG, HGF is expressed mainly in medium and large diameter neurons. Following SNL, HGF expression decreased in L4-L5 DRG neurons (Zheng et al., 2010). HGF in combination with BDNF and NT3, had no impact on DRG sensory neurite outgrowth *in vitro*. In contrast, HGF cooperated with NGF to enhance axonal outgrowth (Maina et al., 1997). Similarly, HGF also cooperates with CNTF positive neurons in supporting the survival and growth of parasympathetic and proprioceptive neurons (Davey et al., 2000). Repeated intramuscular injection of human HGF gene to a crush injured rat showed increased expression of HGF protein and mRNA level in DRGs associated with improvements in function and structure of the crushed nerve (Kato et al., 2005; Boldyreva et al., 2018; Ko et al., 2018).

#### Cardiotrophin-1

Cardiotrophin-1 (CT-1) supports the survival of developing motor neurons *in vivo* and *in vitro* (Pennica et al., 1996; Oppenheim et al., 2001). CT-1 signals by activating the leukemia inhibitory factor, gp130 (LIFRβ/gp130) and the CT-1 α receptor subunit (CT-1Rα) receptor complex (Pennica et al., 1995; Robledo et al., 1997). CT-1 protects animals from progressive motor neuronopathy (PMN), a condition in which mice suffer from motor neuronal degeneration of facial motoneurons and phrenic nerve myelinated axons (Bordet et al., 1999). CT-1 also prevents deterioration in wobbler mice motor neuron disease (MND): paw position, walking pattern abnormalities, intramuscular axonal sprouting and large myelinated motor axons (Mitsumoto et al., 2001), indicating CT-1 may have therapeutic benefits in patients with MND.

#### Neurturin

Neurturin (NRTN) supports embryonic and adult rat midbrain dopaminergic neurons (Horger et al., 1998; Reyes-Corona et al., 2017). NRTN signaling activates Ret tyrosine kinase together with a glycosylphosphatidylinositol (GPI)-linked coreceptor (either GFRα1 or GFRβ2) (Kotzbauer et al., 1996; Golden et al., 1999). NTN^−/−^ mice had loss of GFRα2-expressing neurons from DRG and trigeminal sensory ganglia (Heuckeroth et al., 1999). Neuturin and activated GFRα2 receptor are important for parasympathetic innervation of mucosae (Wanigasekara et al., 2004). NRTN has been shown to upregulate B1 (bradykinin) receptors expressed in isolated nociceptive neurons in mice, indicating a possible influence on pain and inflammation pathways (Vellani et al., 2004).

#### Artemin

Artemin (ARTN) supports the dopaminergic neurons in the rat embryonic ventral midbrain (Baloh et al., 1998b). ARTN operates through GDNF family receptor GFRα3, together with RET tyrosine kinase receptor (Baloh et al., 1998b). After optic nerve injury, ARTN receptor GFRα3 mRNA and protein levels increased within the first week. ARTN and its receptor (GFRα3), offered neuroprotection of injured RGCs through the PI3K-AKT signaling pathway and enhanced optic nerve regeneration in rats (Omodaka et al., 2014). In the PNS, ARTN is expressed in both immature and mature myelinating SCs. After injury, ARTN was highly expressed in the distal nerve segment, indicating that it influences both developing and regenerating peripheral neurons (Baloh et al., 1998b; Ikeda-Miyagawa et al., 2015). In neonatal rat neuron cultures *in vitro*, ARTN supported the survival of sensory neurons derived from the DRG and the trigeminal ganglion (TG) and visceral sensory neurons of the nodose ganglion (NG; Baloh et al., 1998b). *In vivo*, ARTN is expressed in both large and small sensory neurons before and after injury (Baloh et al., 1998b; Wang et al., 2014). Treatment of injured peripheral nerves with ARTN enhanced motor, sensory axon regeneration including functional recovery (Jeong et al., 2008; Wang et al., 2008, 2014; Widenfalk et al., 2009; Zhou et al., 2009; Wong L. E. et al., 2015).

#### Persephin

Persephin (PSP) supports midbrain dopaminergic neuron survival (Milbrandt et al., 1998) in a manner similar to other neurotrophic factors like GDNF and NRN (Baloh et al., 1998a; Milbrandt et al., 1998; Leitner et al., 1999). However, PSP binds efficiently only to GFRα-4 receptors (Enokido et al., 1998). Recent work has suggested that PSP has a neuroprotective effect in animal models of Parkinson’s disease (Yin et al., 2015). In the PNS, PSP supports the survival of motor neurons but not autonomic neurons in the superior cervical ganglion (SCG), sensory neurons, or enteric neurons (Milbrandt et al., 1998).

#### Bone Morphogenetic Proteins

Bone morphogenetic proteins (BMPs) belong to the TGF-β superfamily. BMPs signal using serine/threonine kinasetype I and type II receptors (Ebendal et al., 1998). BMP-2 is expressed in normal sciatic nerve. After injury, BMP-2 was localized at both distal and proximal stumps (Tsujii et al., 2009). BMP-2 improved regeneration of facial nerves and acted as a potential neurotrophic factor (Wang et al., 2007). In contrast, treatment of E10 (mouse embryos) trigeminal neurons with BMP4 *in vitro* resulted in neuronal death, indicating responsiveness of the neurons to BMP4 (Guha et al., 2004). However, BMP-4 promotes the survival of motor neurons and protects neurons from glutamate-induced toxicity (Chou et al., 2013). Treatment with BMP-7 improved recovery in spinal cord injured rats (Chen et al., 2018). In double-ligated sciatic nerves, BMP4 protein was expressed at both the proximal and distal portion of motor axons, indicating BMP-4 proteins were anterogradely and retrogradely transported (Chou et al., 2013). During Wallerian degeneration, BMP-7 expression was increased within proximal and distal injured nerve stumps (Tsujii et al., 2009).

#### Epidermal Growth Factor

Epidermal growth factor (EGF) promotes the proliferation of fibroblast and epithelial cells (Carpenter and Cohen, 1979; Plata-Salamán, 1991; Wong and Guillaud, 2004). EGF-induced neurotrophic action is mediated by activation of EGF receptor (EGFr) and plays a vital role during the development of mouse and rat brain (Yamada et al., 1997; Wong and Guillaud, 2004; Yang et al., 2018). In the adult normal human DRG, Epidermal growth factor receptor (EGFR) is strongly expressed in the small, intermediate size neurons, satellite glial cells and SCs (Huerta et al., 1996). In the sciatic nerve, EGFr mRNA and protein are expressed mainly in both SCs and fibroblasts in rats. After transection injury, EGFr mRNA and protein levels increased in both proximal and distal nerve stumps (Toma et al., 1992). During development, EGF limited the neurite branching in DRG (Explant and dissociated) neurons *in vitro* (Maklad et al., 2009). Interestingly, ablation of EGFr was associated with disorganized sensory innervation in dorsal skin, indicating that EGFr is required for proper cutaneous innervations of the skin (Maklad et al., 2009).

#### Fibroblast Growth Factor (FGF)

Basic fibroblast growth factor (bFGF or FGF2), is a potent growth factor for mesoderm derived cells and acts as a neurotrophic factor to various neural cells. It has been found to enhance hippocampal, cerebral cortical, granule, ciliary ganglion and spinal cord neuron survival. bFGF also promotes neurite extension (Gospodarowicz, 1979; Gospodarowicz et al., 1984; Morrison et al., 1986; Walicke et al., 1986; Unsicker et al., 1987; Hatten et al., 1988; Fujimoto et al., 1997). FGF mainly mediates its trophic action by binding to membrane-bound receptors (FGF receptors) that possess tyrosine kinase activity to generate downstream signal transduction (Imamura et al., 1988; Walicke et al., 1989; Ornitz and Itoh, 2015). In the PNS, both acidic and basic fibroblast factors (aFGF, bFGF) mRNA levels were expressed in small and medium-size DRG neurons (Ji et al., 1995; Acosta et al., 2001). After 3 days of sciatic nerve injury, aFGF(FGF1) mRNA levels increase in DRG neurons but bFGF mRNA levels were upregulated in most DRG neurons but declined after 1 week (Ji et al., 1995). In addition, FGF 2 and 7 were also increased in lumbar DRG neurons but FGF 13 levels decreased after injury (Li et al., 2002). In the sciatic nerve, FGF 2 and FGF receptor (FGFR1-3) mRNA expression increased after injury (Grothe et al., 2001). Local treatment with bFGF after injury increased the number of regenerating axons. FGF receptor expression increased in the proximal and distal segments (Fujimoto et al., 1997; Archer et al., 1999; Grothe and Nikkhah, 2001; Namaka et al., 2001). Interestingly, FGF-2 overexpression generated SC proliferation, doubled the number of regenerating axons and enhanced remyelination after sciatic crush in mice (Jungnickel et al., 2006). Treatment with recombinant human FGF-2 (rhFGF-2) to a mental nerve crush injured rats had improved regeneration and sensory functional recovery (Lee et al., 2017).

#### Osteopontin

Osteopontin (OPN) is a secreted phosphoprotein that interacts with receptor α_v_β_3_ integrin (Liaw et al., 1995). OPN plays an important role in rat brain development (Shin et al., 1999; Lee et al., 2001). Furthermore, OPN expression was increased in pyramidal neurons in Alzheimer’s disease (AD) brain in comparison to age-matched controls (Wung et al., 2007). In the PNS, OPN is expressed mainly in the larger sized DRG and TG neurons (Ichikawa et al., 2000). After injury, OPN was expressed in the degenerating distal nerve stump during the first day but was downregulated at day 14. Later stage after axotomy, SC-OPN was re-expressed in regenerating crushed nerves but not in permanently transected nerves (Jander et al., 2002; Wright et al., 2014).

#### Insulin

Insulin acts as a growth factor that supports the survival and synaptic plasticity of neurons (Fernyhough et al., 1993; Toth et al., 2006; Hoybergs and Meert, 2007; McNay et al., 2010; Singh et al., 2012b). Insulin receptor (IR) mRNA is regulated during postnatal peripheral nerve development (Shettar and Muttagi, 2012). In dissociated neurons *in vitro*, insulin signaling regulates neurite outgrowth (Fernyhough et al., 1989, 1993; Govind et al., 2001; Choi et al., 2005; Singh et al., 2012b). Neurons may also have the capacity to synthesize insulin (Devaskar et al., 1994; Rulifson et al., 2002). In adult DRG neurons, IR mRNA and protein levels were higher in small caliber neurons and sciatic nerves (Sugimoto et al., 2000, 2002). Similarly, insulin receptor subunit β (IRβ) expression was increased in sensory neurons after sciatic nerve crush injury (Xu et al., 2004) and has been identified in dermal fibers of mouse foot pads (Guo et al., 2011). In injured sciatic nerves, systemic insulin administration enhanced reinnervation of foot interosseous endplates associated with enhanced functional recovery (Xu et al., 2004). Intrathecal insulin increased calcitonin gene related peptide (CGRP) expression in DRG neurons, enhanced functional recovery of sensation and increased axon regrowth rate identified by the pinch test following sural nerve crush (Toth et al., 2006). In diabetic rats, near sciatic nerve insulin treatment enhanced local motor conduction velocities but also increased the percentage of small (≤9.0 μm diameter) myelinated fibers within nerves exposed to it (Singhal et al., 1997). Local sub-hypoglycemic insulin has had additional impacts in diabetic models including reversal of axon atrophy after intrathecal injection, enhanced epidermal axon regrowth following local injection and improvements in neuropathy from intranasal injection (Brussee et al., 2004; Guo et al., 2011; De la Hoz et al., 2017).

## Electrical Stimulation and Axon Regeneration

One of the most robust empiric approaches, now with evidence of efficacy in humans, has been post-injury exogenous electrical stimulation. In work pioneered by Brushart, Gordon, Verge, Chan and others (Brushart et al., 2002; Geremia et al., 2007; Gordon et al., 2008, 2010; McLean et al., 2014; Wong J. N. et al., 2015), brief post-injury electrical stimulation enhanced the regrowth of motor and sensory axons (Al-Majed et al., 2000a; Gordon et al., 2007). While its full capabilities and exact mechanisms are not fully characterized, they include a retrograde ramp-up of regeneration-associated genes and the action of BDNF.

Work by Gordon et al. established that post-injury stimulation at 20 Hz for 1 h offered an impressive impact on regeneration. Interestingly this is a very specific outcome limited to a well-circumscribed paradigm but not effective with other tested forms of Electrical stimulation (ES). For this review, most of the citations here refer to this specific approach. ES increased the speed and accuracy of axonal regeneration and re-innervation (Al-Majed et al., 2000b; Brushart et al., 2002; English et al., 2007) and benefitted both sensory and motor re-innervation (Al-Majed et al., 2000b; Brushart et al., 2002; Geremia et al., 2007; Gordon et al., 2009; Singh et al., 2012a). In adult DRG neurons *in vitro* plated over stimulating microelectrodes, ES accelerated early neurite outgrowth (Singh et al., 2012a). Brief electrical stimulation then applied *in vivo* to the proximal injured site in mice enhanced regrowth of axons across transection sites (Singh et al., 2012a). ES accelerated the return of reflex foot withdrawal and contractile force in re-innervated leg muscles (Nix and Hopf, 1983; Pockett and Gavin, 1985). ES also increased the numbers of regenerated axon density and diameter after 8 weeks of surgery (Haastert et al., 2011), enhanced myelination and angiogenesis (Lu et al., 2008; Deng et al., 2018). In post-surgical patients that had sustained complete digital nerve transection there were greater improvements in sensory re-innervation following ES (Wong J. N. et al., 2015).

ES facilitates myelination through impacts on SC polarization and BDNF (Wan et al., 2010). Along these lines, delayed brief ES increased expression of myelin basic protein (MBP) and promoted re-organization of the node of Ranvier coinciding with the early reappearance of re-myelinated axons (McLean et al., 2014). ES also accelerated the removal of myelin debris and promoted more vigorous clearance of activated macrophages from the demyelination zone (McLean et al., 2014).

In sensory neurons, enhanced BDNF immunoreactivity expression was identified after ES (Alrashdan et al., 2010). Al-Majed et al. (2000a) demonstrated that 7 days after femoral nerve transection and stimulation the mRNA expression of BDNF and its receptor TrkB level had a two-fold rise within rat femoral motor neurons. In additional work, ES promoted the release of NGF from cultured SCs through calcium influx (Huang et al., 2010). ES is associated with rises in neuronal calcium content (Singh et al., 2012b) a change that might correlate with additional mechanisms of the ES response such as rises in regeneration related molecules including tubulin, Sonic hedgehog (Shh) and GAP43 mRNA (Singh et al., 2015).

## Resurrection of Developmental Molecules in Regeneration

An important regeneration theme is the redeployment of developmental related molecules for new roles during regrowth. The netrin-Deleted in Colorectal Cancer (DCC)-Unc5H interactions are an important example.

Netrins belong to an evolutionarily conserved and developmentally important family of laminin-related proteins (Sun et al., 2011). Netrin-1 receptors include two main families: DCC, comprising DCC and neogenin, and the uncoordinated gene 5 (UNC-5) proteins (Keino-Masu et al., 1996; Ackerman et al., 1997; Leonardo et al., 1997). Ligation of DCC and UNC4H2 receptors by extracellular netrin-1 inhibited apoptosis. DCC and UNC5H2 are also called “dependence receptors” that trigger either survival or apoptotic signals depending on whether netrin-1 is respectively present or absent (Mehlen and Mazelin, 2003; Mehlen and Tauszig-Delamasure, 2014). Netrin-1 up-regulation is important for neuronal navigation (Jiang et al., 2003; Cirulli and Yebra, 2007; Mehlen et al., 2011). Netrin-1 and DCC have specifically been linked to neural crest cell migration (Jiang et al., 2003).

Netrin-1 and its receptor proteins are involved in axonal guidance in *C. elegans* (Serafini et al., 1994; Leonardo et al., 1997) and act as a cue that is bifunctional and attracts or repels different axons. It attracts commissural axons using the DCC receptor and repels others through Unc5 receptors (Kennedy et al., 1994; Moore et al., 2007; Briançon-Marjollet et al., 2008). Moreover, its repulsive guidance may specifically be involved in DRG sensory axon fate during development (Watanabe et al., 2006; Masuda et al., 2008). In the CNS, netrin-1 is also expressed by oligodendrocytes and inhibits regeneration of adult CNS neurons that express Unc5H2 (Manitt et al., 2009). Netrin-1 may also have direct impacts on axon growth and branching (Dun and Parkinson, 2017; Boyer and Gupton, 2018). For example, netrin guides RGC axons as they navigate the visual pathway (Deiner and Sretavan, 1999) but also targets arborization of mature RGC axons. This involves DCC-dependent increases in presynaptic differentiation and dynamic branching (Manitt et al., 2009).

In the PNS, netrin-1 receptors are expressed in sensory and motor neurons, SCs and axons both intact or after injury (Park et al., 2007). DCC receptors and Unc5H2 receptor are expressed in glial cells, particularly in proximal nerve stumps following peripheral nerve injury (Webber et al., 2011). Knockdown of DCC locally, using an siRNA approach directed at the proximal stump of a transected nerve trunk impaired SC activation and outgrowing migration, and was associated with secondarily attenuated regeneration. In contrast, similar local knockdown of Unc5H2 receptors enhanced SC outgrowth and follow on axon regeneration. The overall findings indicated that the netrin-DCC receptor interaction is redeployed from development for use during adult axon regeneration. There were, however, differences between development and adult regeneration. In the former, the specific attractive netrin-DCC and repulsive netrin-UnC5H interactions are among growing neurons. In the adult, this machinery is subsumed by reactive SCs but with the same direction of impact. Since axon-SC interactions are intimate and essential for overall nerve regrowth, knockdown of the Unc5H2 “brake” ultimately benefitted axon regrowth, following along outgrowing SCs. Interestingly, injury itself upregulated DCC receptors and down-regulated Unc5H2 receptors providing facilitation for regrowth. Similarly, knockdown of DCC upregulated its reciprocal Unc5H2 receptor. Taken together, not only was there acquisition of attractive and repellant developmental neuronal molecules by SCs in the adult, their relationship also remained reciprocal. Exogenous netrin-1 peptides added to neurons *in vitro* or sciatic nerves *in vivo* did not impact regeneration, likely indicating sufficient endogenous levels to activate these receptors (Webber et al., 2011). However, higher concentrations of exogenous netrin-1 in adult DRG explants and dissociated DRG culture may inhibit neurite outgrowth (Park et al., 2007).

## Growth Cone GTPases: RhoA and Rac1

Local growth cone molecules may be the final arbiters over whether extension or advancement of axons occurs or whether they retract and withdraw. Among these molecules, a prominent role for the Rho family GTPases exists. RhoA GTPase and its downstream effector Rho-kinase (ROCK) signal an inhibitory pathway involved in cellular growth, differentiation, migration and survival (Mueller et al., 2005). RhoA is activated by GTP binding. RhoA/ROCK activation is involved in growth cone collapse and reduced axonal outgrowth in CNS neurons (Lehmann et al., 1999). Moreover, RhoA/ROCK activation has been detected in both spinal cord and optic nerve injury (Lehmann et al., 1999; Fu et al., 2016). After spinal cord injury, expression of RhoA was increased in neurons, astrocytes and oligodendrocytes (Dubreuil et al., 2003), indicating an important role in the inhibition of CNS regeneration (Hu and Selzer, 2017). Thus, the ROCK inhibitor, Y27632 administered after spinal cord injury was associated with new axon sprouts in the gray matter distal to injury and improved functional recovery (Chan et al., 2005).

RhoA/ROCK impacts PNS axon regeneration. In the PNS, RhoA and ROCK1 mRNA and proteins are expressed in the dorsal root ganglion (DRG) neurons, axons and SCs of the sciatic nerve and upregulated after injury (Terashima et al., 2001; Cheng et al., 2008). Moreover activated RhoA GTPase was upregulated in proximal stumps of transected nerve trunks (Cheng et al., 2008). RhoA protein was also increased in motor neurons after mouse sciatic nerve injury (Hiraga et al., 2006; Joshi et al., 2015). Pharmacological inhibition of RhoA-ROCK, using the small molecule inhibitor HA1077 promoted neurite and axonal outgrowth of DRG neurons *in vitro*. Furthermore, when the ROCK inhibitor was applied to the tip of the sciatic nerve injury site, the number of outgrowing axons and associated SCs was enhanced (Cheng et al., 2008). RhoA GTPase also is involved in growth cone behaviour of PNS neurons. Specifically, application of a ROCK inhibitor induced growth in sensory neuron growth cones (Guo et al., 2014). Hiraga et al. (2006), noted that the ROCK inhibitor (fasudil) increased amplitudes of distally evoked compound muscle action potentials after axonal injury. Along with these physiological benefits, the agent increased numbers and caliber of regenerating axons indicating a role in promoting axon maturation through ROCK inhibition.

## A Role for Tumor Suppressors in Neurons: PTEN Is An Intrinsic Blocker of Axon Regrowth

PTEN is a tumor suppressor that converts phosphatidylinositol (3,4,5)-triphosphate (PIP3) into phosphatidylinositol (4,5)-biphosphate (PIP2). On inactivation of PTEN, PIP3 accumulates, thereby phosphorylation activating Akt whereas pAkt subsequently inhibits GSK3β, itself an inhibitor of axon growth. Knockdown of PTEN and activating the PIP3/Akt pathway is closely linked to proliferation, cell survival, increased cell size and epithelial polarity. Mutations in PTEN are found in malignant glial brain tumors (Ali et al., 1999; Broderick et al., 2004). Loss of heterozygosity of PTEN is observed in human malignancies especially endometrial and ovarian cancer, late-stage metastatic tumors and others (Li and Sun, 1997). Inactivation of a single PTEN allele increases cell proliferation and cell survival and reduces apoptosis (Di Cristofano et al., 1999; Podsypanina et al., 1999). Accordingly, PTEN has many roles in the nervous system during development and adulthood (Kath et al., 2018). Recent studies have demonstrated that neurotrophin-related growth and differentiation is specifically inhibited by PTEN overexpression (Musatov et al., 2004). Conditional deletion of PTEN in the developing hippocampus and cortex is associated with neuronal hypertrophy and behavioral alterations that model human autism (Kwon et al., 2006). On the other hand, PI3K activation regulates neuronal differentiation, survival migration, extension and guidance (Brunet et al., 2001; Rodgers and Theibert, 2002; Arimura and Kaibuchi, 2005; Chang et al., 2006).

PI3K/Akt signals are expressed and activated during axon regeneration. For example, knockdown of PTEN by siRNA increased neuronal polarity and axonal outgrowth in hippocampal neurons *in vitro* (Jiang et al., 2005). PTEN deleted mice had increased RGC survival and extended robust long-distance axon regeneration after 14 days of optic nerve injury. Inactivation of PTEN leads to activation of PI3K, pAkt and mammalian target of rapamycin (mTOR) signaling in CNS neurons. Studies from several models (*C. elegans* to mammalian neurons) have identified a role for PI3K in asymmetric signaling and its impact on orienting polarized outgrowth during axonogenesis (Yoshimura et al., 2005; Adler et al., 2006). PTEN knockdown through enhanced mTOR activity increased RGC survival and axon regeneration whereas rapamycin blocked mTOR activity and attenuated regeneration. Further, axotomy in RGCs markedly reduced pS6 levels possibly accounting for the limited CNS regeneration after crush injury (Park et al., 2008). S6 ribosomal kinase 1 is targeted by the mTOR pathway, and its phosphorylation is indicative of mTOR activity (Kim et al., 2016). Similarly, PTEN deleted mice had improved repair of the corticospinal tract (CST) of spinal cord injury mice (Geoffroy et al., 2016). Inhibition of PTEN improves outcome in experimental spinal cord injuries, with lesser motorneuron death, greater tissue sparing and smaller cavity formation (Walker et al., 2012).

**Figure 2:**
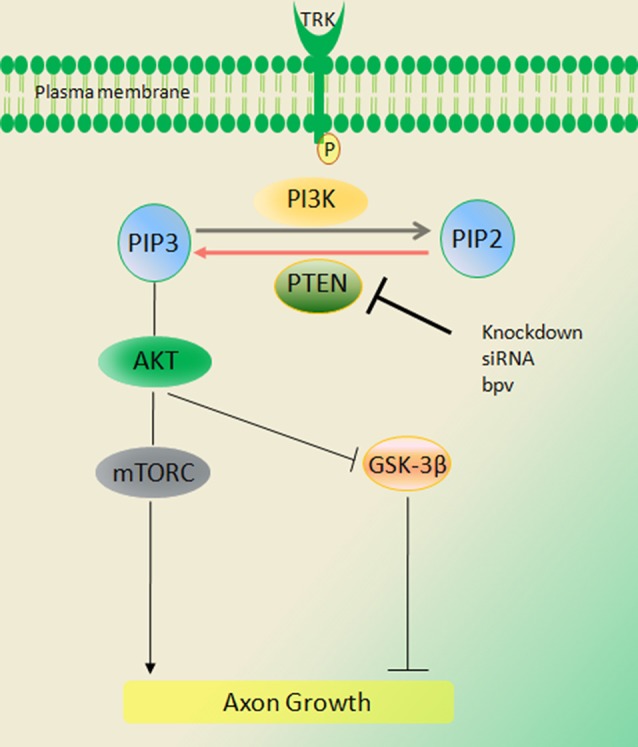
Signaling of the phosphatase and tensin homolog deleted on chromosome ten (PTEN)/PI3K/Akt pathway. PTEN converts phosphatidylinositol (3,4,5)-triphosphate (PIP3) into phosphatidylinositol (4,5)-bisphosphate (PIP2), thus inhibiting Akt activation and peripheral nerve regeneration. Activation of PI3K and Akt signals through deletion of PTEN [BPV (pic) or siRNA] increases regeneration of axons in injury. These impacts appear to be independent of mammalian target of rapamycin (mTOR) but require the activity of PI3K and Akt (Duraikannu, original illustration).

PTEN knockdown is associated with substantial benefits following peripheral nerve injury. PTEN is expressed widely in sensory and motor neurons but there is intense expression among small caliber IB4 nonpeptidergic DRG neurons. Paradoxically its levels rise after nerve injury. PTEN is also expressed in SC and in regrowing injured axons (Christie et al., 2010). Local inhibition using either the pharmacological inhibitor BpV(pic) or siRNA knockdown enhances axon outgrowth as evaluated in both *in vitro* and *in vivo* analysis (Figure 2; Christie et al., 2010). PTEN knockdown using siRNA was accomplished without a viral vector. In the PNS these impacts appear to be independent of mTOR but require the activity of PI3K and Akt.

PTEN may influence distal regenerative events within growing axons through central rather than peripheral modulation of cellular machinery. For example, local exposure of adult growth cones to gradients of the PTEN inhibitor, BpV (pic) did not have significant impacts on growth cone turning, unless combined with a local growth factor. However, when inhibitory gradients were instead directed at the perikarya of *in vitro* adult sensory neurons, there was a striking rise of distal outgrowth in distal branches growing in directions unrelated to the inhibitory gradient. These data suggest that PTEN modulation is a central deterministic signal that instructs distal growth cone behavior (Christie et al., 2010).

In a mouse spinal muscular atrophy (SMA) model, knockdown of PTEN rescued defects in axon length, growth cone structure and overall survival (Ning et al., 2010). In a chronic diabetic neuropathy model, with documented regenerative failure, PTEN levels in motor and sensory neurons were upregulated (Singh et al., 2014). In keeping with this finding, PTEN knockdown rescued the regenerative deficit.

## A Further Tumor Suppressor and Its Impact on Adult Neurons: Retinoblastoma 1

The retinoblastoma tumor suppressor Retinoblastoma 1 (Rb1) operates at the core of the cell cycle pathway. Its mutations are associated with childhood retinoblastoma tumors. Rb1 is linked to neuronal fate, regulating proliferation and migration of neuronal progenitors during brain development (Slack et al., 1998; Ferguson et al., 2002, 2005; McClellan et al., 2007; Andrusiak et al., 2010). In the canonical pathway, Rb1 regulates cell cycle progression by binding and signaling through the E2F family of transcription factors. The operational status of Rb1 protein depends on its phosphorylation status, in turn mediated by the cyclin/cyclin-dependent kinase (CDK) complex (Giacinti and Giordano, 2006).

Deletion of Rb1 is a frequent and early molecular hallmark of cancer. Specifically, individuals with germ-line Rb1 mutations are at risk of developing trilateral retinoblastoma, a pediatric intracranial neuroblastic tumor (Jakobiec et al., 1977; Marcus et al., 1998). Rb null mice die as embryos by E15 from hematopoietic and neurological abnormalities linked to the failure of cells to permanently withdraw from the cell cycle (Clarke et al., 1992; Jacks et al., 1992; Lee et al., 1992). Conditional deletion of Rb1 in the embryonic retina display ectopic division and apoptosis of developing retinal transition cells (MacPherson et al., 2004; Zhang et al., 2004a). Rb1 functions within RGC axons, such that its absence is associated with retinal and midline pathfinding errors, leading to aberrant tectal innervation. In sensory ganglia, extensive loss of sensory neurons and expression of TrkA, B neurotrophin receptors was associated with Rb1 deletion during development (Lee et al., 1992).

Rb deletion induces cell cycle re-entry in several systems (Sage, 2012), including mouse embryonic fibroblasts (MEFs; Sage et al., 2003), mammalian muscle cells (Zacksenhaus et al., 1996; Pajcini et al., 2010) and adult cortical neurons (Andrusiak et al., 2012). In contrast, loss of Rb is capable of driving mutated SC growth through a signaling pathway distinct from PI3-AKT-mTOR and using an E2F-independent mechanism (Collins et al., 2012).

As discussed, previous studies indicate that the PI3K/Akt signaling pathway influences axon outgrowth and neuronal plasticity and that these roles overlap with protection and survival. In postmitotic adult injured neurons, Rb1 may influence the downstream PI3K-Akt pathway on growth, a pattern resembling the impact of PTEN (Christie et al., 2014). Following sciatic nerve injury, Rb was robustly expressed in neurofilament labeled DRG neurons and axons, despite its role as an inhibitor of sensory neuron growth after injury. Like PTEN, the Rb1 protein paradoxically rises following injury and may also operate downstream of Raf-MEK and the PI3K-Akt pathway. *In vitro* knockdown of Rb using siRNA increased neurite outgrowth and length in dissociated adult sensory neurons (Christie et al., 2014). As in the PTEN knockdown studies and subsequent adenomatous polyposis coli (APC) work described below, the approach used nonviral methods to achieve knockdown. In addition, silencing of Rb protein enhanced neurite branching in both uninjured and injured DRG neurons. These actions were abrogated with concurrent knockdown of the Rb1 effector, E2F1. E2F1 operates as a divergent transcription factor and stimulates transcription and neuronal plasticity. In adult neurons, knockdown of Rb1 was not associated with cell death with no impact on the expression of activated caspase-3 or DNA damage markers (phosphohistone H2A.X). *In vivo* local knockdown at a nerve crush site enhanced regeneration of axons and promoted functional recovery in injured mice (Christie et al., 2014).

## BRCA1 Protects Regenerating Neurons

Breast cancer susceptibility protein 1 (BRCA1), a tumor suppressor, plays a critical role in DNA repair and CNS development (Miki et al., 1994). BRCA1 is expressed in proliferating embryonic and adult neural stem cells (Korhonen et al., 2003). Deleting BRCA1 in the CNS, results in various abnormalities in brain development and overall brain volume is severely reduced, apparent in the neocortex, cerebellum, and olfactory bulbs (Gowen et al., 1996; Pulvers and Huttner, 2009; Pao et al., 2014). BRCA1 is also involved in rat RGC neuron survival and DNA repair after exposure to ionizing radiation *in vitro* (Wang et al., 2018). In spinal cord injury, BRCA1 is highly expressed in spinal microglia (Noristani et al., 2017). In the PNS, BRCA1 is expressed in DRG, sciatic nerve and SCs in adult rat and after injury, is expressed at high levels in both proximal and distal nerve. BRCA1 expression was identified in injured SCs, neuronal satellite cells and axons and it translocated to neuronal nuclei. Interestingly, BRCA1 supported the regenerative phenotype in neurons such that its knockdown was associated with a decrease in neurite outgrowth and reduced branch length of injured sensory neurons *in vitro* (Krishnan et al., 2018b). In SCs BRCA1 depletion impaired SC proliferation. BRCA1 modulates oxidative stress in injured sensory neurons and SCs (Krishnan et al., 2018b). It appears critical in modulating DNA repair by preserving DNA integrity in neurons, particularly after injury through its nuclear enrichment. The role of BRCA1 in sensory neurons has taught us that DNA repair may be an intrinsic element of their acquisition of a regenerative phenotype. In addition to BRCA1, adult peripheral neurons constitutively express additional DNA repair molecules, such as 53BP1, important for their ongoing wellbeing.

## APC and Its β-Catenin Partner

The parallel but thus far largely unconnected impact of two critical tumor suppressor pathways, PTEN and Rb1, on post-mitotic adult sensory neurons was remarkable. BRCA1 appears to have a different operational mandate in neurons. Given all of these findings, however, we hypothesized that exploitation of a range of tumor-related pathways might be a general property of regenerating adult neurons. Along these lines, we chose to study yet an additional tumor suppressor pathway, APC and its β-catenin pathway, important culprits in the development of colorectal tumors.

Wnt/β-catenin signaling plays a significant role in neurodevelopment and neuronal plasticity (Tawk et al., 2011). The Wnt/β-catenin pathway contributes toward oligodendrocyte and SC myelination; the expression of myelin genes, SC migration, and their proliferation in the PNS (Tawk et al., 2011). In Wnt signaling, β-catenin is an important multifunctional transcriptional protein, binding to APC and GSK3β. A remarkable feature of β-catenin protein is that it promotes cell proliferation and resistance to apoptosis (Clevers, 2006; Shelton et al., 2006). APC is a binding partner to β-catenin that results in a destruction complex involving proteasomal degradation and transcriptional inhibition (Kimelman and Xu, 2006). Through this interaction, APC is involved in proliferation, apoptosis, cell adhesion, and migration (Hanson and Miller, 2005). APC activities also play a vital role in both the developing as well as adult nervous system (Bhat et al., 1994). APC is expressed in neurites of neuroblastoma cells and cortical neurons (Morrison et al., 1997a,b). Loss of APC enhances β-catenin accumulation in the nucleus with its transcriptional partner T cell factor/lymphoid enhancer factor (TCF/LEF).

β-catenin expression influences neural proliferation and neuronal differentiation (Patapoutian and Reichardt, 2000) and it is involved in hippocampal neurogenesis (Peng et al., 2009). Furthermore, β-catenin phosphorylation at residue Y654 and Y142 and its nuclear localization increases axon growth and branching in hippocampal neurons through TCF4/β dependent transcription (David et al., 2008). In post-mitotic neurons, it is involved in dendritogenesis, synaptogenesis and synaptic formation (Peng et al., 2009). Both Wnt and β-catenin combined regulate synaptic plasticity and axonal growth. In CNS neurons, N and C-terminal domains of β-catenin are involved in cell-cell adhesion and promotion of axonal branching (Elul et al., 2003). Increased expression of β-catenin and Wnt were observed after spinal transection in adult zebrafish and correlated with axonal regeneration and improved functional recovery (Strand et al., 2016). With spinal cord lesions, Wnt/β-catenin signaling also regulates collagen type XII alpha 1 chain (col12a1) transcription and synthesis of Collagen XII by non-neuronal cells such as fibroblasts for the extracellular matrix (ECM). These actions promoted axon regeneration and functional recovery (Wehner et al., 2017). Knockdown of β-catenin showed a delay in axonal sorting whereas gain-of-function of  β-catenin mutations resulted in accelerated sorting (Grigoryan et al., 2013).

**Figure 3:**
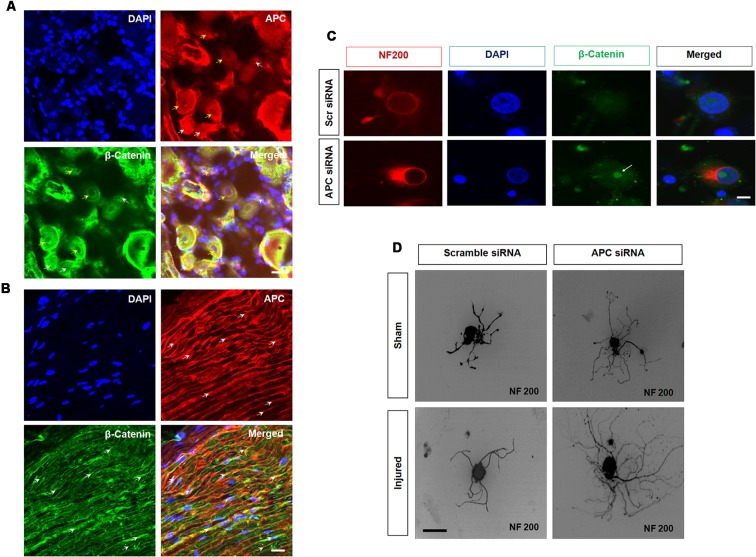
Adenomatous polyposis coli (APC) and β-Catenin in sensory neurons. **(A,B)** APC and β-Catenin co-localize in the dorsal root ganglia (DRG) and sciatic nerve. **(A)** APC is expressed in β-catenin positive DRG neurons. Importantly, APC is prominently co-localized with β-Catenin in a subset of small (white arrow) and medium size (yellow arrow) uninjured DRG neurons. **(B)** Longitudinal section of uninjured sciatic nerve. APC (red, white arrow) and β-catenin (green, white arrow) showing colocalization. **(C)** Z stacks confocal picture demonstrate the nuclear localization of β-catenin in cultured DRG neurons with APC knockdown when compared to those exposed to scrambled control siRNA *in vitro*. Scale bar = 50 μm. **(D)** Silencing APC by siRNA increased neurite outgrowth in cultured DRG neurons when compared to scrambled siRNA in both sham sciatic injury and sciatic axotomy pre-conditioning injury. Scale bar = 50 μm (Duraikannu, original illustration).

**Figure 4:**
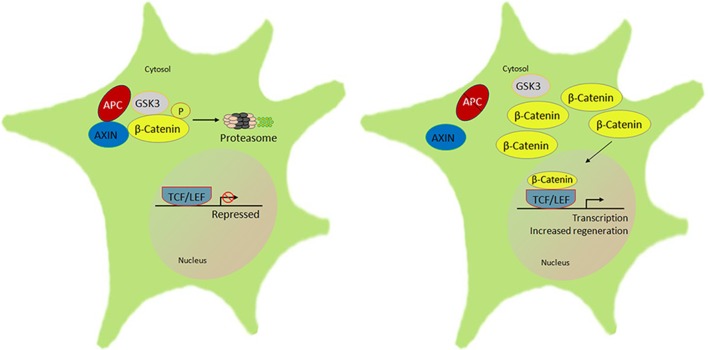
Schematic of the β-catenin-lymphoid enhancer-binding factor/T cell factor (LEF/TCF) signal pathway that regulates peripheral neuron regeneration by APC knockdown. There is normally interaction of APC with AXIN and GSK3 that phosphorylates β-catenin to be rapidly degraded by the ubiquitin proteasome. In the absence or knockdown of APC signal with an altered AXIN-GSK3β complex, β-catenin accumulates and forms a complex with LEF/TCF in the nucleus, which initiates transcription of downstream target genes, in turn to promote regeneration (Duraikannu, original illustration).

In adult DRG sensory neurons, β-catenin was expressed widely among all sensory neurons including neuronal nuclei, and cytoplasm and was also identified in perineurial satellite cells. Axonal injury results in reduced β-catenin expression in neuronal nuclei, satellite cells and sciatic nerves. In contrast, its binding partner APC was reciprocally increased following axotomy injury. Notably, after injury, expression of APC was prominent within slower growing small nonpeptidergic IB4 DRG neurons and SCs, in a remarkable similarity to PTEN expression. Furthermore, the intensity of APC expression increased in motor neurons and regenerating axons. The rises of APC, considering its role to inactivate transcriptional-related targets, indicated that it might act as a regenerative “brake” on neurons. Indeed, both the APC and β-catenin partners are combinatorially expressed in DRG neurons and their axon branches (Figure 3). Overexpression of APC interacts with β-catenin to enhance proteasomal degradation and attenuate transcription. In sensory neurons, knockdown of APC increased β-catenin nuclear accumulation, was associated with upregulation TCF and lymphoid enhancing binding factor (LEF) transcription factors (Figure 4). In addition, knockdown of APC in adult DRG neurons increased neurite outgrowth of both uninjured and preconditioned neurons (Duraikannu et al., 2018). Narciso et al. (2009), showed that β-catenin expression increased in Galectin-3 knockout mice associated with enhanced neuronal survival and axon regeneration after traumatic nerve lesions. Furthermore, interactions between β-catenin and TCF3 were required for SC myelination *in vivo* (Tawk et al., 2011). Inhibition of β-catenin and TCF activity impaired neurite outgrowth in DRG neurons *in vitro* (Duraikannu et al., 2018). *In vivo*, local knockdown of APC following nerve trunk crush injury using siRNA increased the repopulation of myelinated axons and was associated with improved indices of functional recovery. In keeping with the reciprocal actions of these partners, knockdown of APC led axons and SCs to express higher levels of β-catenin *in vivo*. Moreover, β-catenin appears to be required for SC proliferation *in vitro*, a critical partnering step in nerve regeneration (Narciso et al., 2009).

Overall, manipulation of the APC-β-catentin pathway has provided yet another example of how plasticity molecules are expressed in apparently stable and hard-wired neural systems. However, after injury, they play a role in supporting the plasticity essential for regeneration. In the case of APC, knockdown of its inhibitory impact releases β-catenin transcriptional activation required to support repair and outgrowth. Like PTEN and Rb1, APC is a new and important tumor suppressor in neurons that normally suppresses their growth. Whether this approach offers synergy with other tumor suppressor pathways is unclear at this time.

## Conclusions

The primary goal of many endpoints in the clinical treatment of nerve injury and neuropathies is to identify improved functional recovery. Here, we identify how that may be accomplished through the actions of neuronal growth factors but more recently by manipulating the intrinsic growth properties of adult neurons. These approaches operate downstream of growth factors, that have the potential for off-target actions and that have impacts limited to specific subtypes of neurons. Exogenous ES activates neuronal regenerative progress through a “brute force” reset of intrinsic neuron properties that recapitulates their injury response. By doing so, the approach exploits the known property of previously injured neurons to ramp up their regenerative machinery, the “preconditioning” response. Resurrection of developmental pathways to enhance regeneration in adults likely has more room for investigation. Recognition that the array of receptors and expression patterns of neurons differs between development and adulthood is essential toward further understanding of their potential role. Re-appropriation of pathways used by neurons in development by glial cells, as described with netrin-DCC-Unc5h, is an important example of this.

Here, we focus most of this review on novel intrinsic pathways that have remarkable impacts on regrowth, downstream from growth factor receptors. That all three pathways described here influence the development of neoplasms should not be seen as a disincentive for further consideration. It is unlikely that anatomically restricted administration and temporary use of knockdown during regeneration would replicate the complex and multistage prolonged processes required for oncogenesis. In the case of BRCA1, ongoing DNA repair during neuron reprogramming toward a regenerative state may be essential. Further work is required over how the growth pathways presented here, and very likely additional neuron growth pathways interact. It is not known whether they operate synergistically with a downstream common impact on PI3K-pAkt signaling, an unequivocal and critical pathway that supports regrowth. It is also yet to be determined whether manipulation of intrinsic growth pathways might be coupled with added growth factors. Finally, the use of nonviral siRNA delivery has been an important part of regenerative studies using *in vivo* models. The elimination of viral delivery constructs will prove to be a major advantage to its use in humans and builds on the recognition that there is considerable extracellular trafficking capability in using siRNA. We have, for example, shown that siRNAs are routinely taken up by distal axons and retrogradely transported to their perikarya to knockdown “central” cellular gene expression. Improved approaches to enhance the stability of applied siRNAs and their access to penetrate the blood-brain barrier will be very welcomed.

## Author Contributions

AD: collated background literature, prepared the figures and wrote initial and revised versions of the manuscript prior to submission. DZ: the scope of the review article, wrote portions of the manuscript, and edited all versions prior to submission, including the final submitted version. AK and AC: contributed to the experimental work reviewed in this article, reviewed the intellectual content of the article and edited the final version.

## Conflict of Interest Statement

The authors declare that the research was conducted in the absence of any commercial or financial relationships that could be construed as a potential conflict of interest.
